# Development of Self-Emulsifying Drug Delivery Systems (SEDDSs) Displaying Enhanced Permeation of the Intestinal Mucus Following Sustained Release of Prototype Thiol-Based Mucolytic Agent Load

**DOI:** 10.3390/molecules27144611

**Published:** 2022-07-19

**Authors:** Ahmad Malkawi, Nasr Alrabadi, Razan Haddad, Azhar Malkawi, Khaled Khaled, Airemwen Collins Ovenseri

**Affiliations:** 1 Faculty of Pharmacy, Cyprus International University, Nicosia 99258, Cyprus; khaledhussam97@gmail.com (K.K.); acollins@ciu.edu.tr (A.C.O.); 2 Department of Pharmaceutical Sciences, Faculty of Pharmacy, Isra University, Queen Alya Airport Street, Amman 11622, Jordan; 3 Department of Pharmacology, Faculty of Medicine, Jordan University of Science and Technology, Irbid 22110, Jordan; nnalrabadi@just.edu.jo; 4 Department of Pharmaceutical Technology, Faculty of Pharmacy, Jordan University of Science and Technology, Irbid 22110, Jordan; rhhaddad17@ph.just.edu.jo (R.H.); azharmalkawi5@gmail.com (A.M.); 5 Department of Pharmaceutics and Pharmaceutical Technology, Faculty of Pharmacy, AL-Ahliyya Amman University, Amman 19328, Jordan

**Keywords:** controlled release, ionic complexation, mucodiffusion, porcine intestinal mucus, rheological parameters, SEDDS

## Abstract

In this study, mucoactive self-emulsifying drug delivery systems (SEDDSs) based on sustained release of *N*-acetylcysteine (NAC) were developed for providing effective intestinal mucopermeation. Polymeric ionic complexes of NAC were formed with polyethyleneimine (PEI), Eudragit E 100, and Eudragit RS 100 and loaded into a novel SEDDS. The SEDDSs exhibited a stable average size of 75 ± 12 nm (polydispersity index (PDI) < 0.3) and showed a rise in the zeta potential from −17.31 mV to −7.72 mV. On Caco-2 cells, SEDDSs at 1–3% were non-cytotoxic. An average of 91.8 ± 5.4% NAC was released from SEDDSs containing Eudragit E 100 (*p* ≤ 0.05) and Eudragit RS 100 (*p* ≤ 0.001) complexes at a significantly slower rate within 80 min, whereas the SEDDS containing PEI released NAC in a matter of seconds. Similarly, the SEDDS complexes revealed a time-dependent reduction in mucus dynamic viscosity of 52.6 ± 19.9%. Consequently, as compared with a blank SEDDS, mucodiffusion revealed about 2- and 1.8-fold significantly greater mucopermeation of SEDDSs anchoring Eudragit E 100–NAC and RS 100–NAC complexes (*p ≤* 0.05), respectively. The mucoactive SEDDSs, which steadily released NAC while permeating the mucus, were linked to a significantly increased mucopermeation in vitro as a result of optimal mucolytic targeting.

## 1. Introduction

Self-emulsifying drug delivery systems (SEDDSs) are a platform oral dosage form, which allows for the solubilization of a wide range of pharmaceuticals. When these systems come in contact with gastrointestinal (GI) fluids, on the one hand, they spontaneously create an oil-in-water emulsion, providing a suitable method of drug administration delivered via the intestinal mucosal route [1,2]. Intestinal epithelial absorption of drugs hindered by a covering mucus gel layer, on the other hand, poses a problem. The challenging steric impediment of the intestinal mucus encountered by the intraluminal diffusing SEDDSs to the absorptive epithelium may contribute to the loaded essential drug’s poor systemic bioavailability [3]. To penetrate the mucus barrier, two main strategies can be presented: inducing alterations within SEDDS physicochemical properties as a passive strategy and developing SEDDSs that actively interact with mucus [4]. Concerning passive strategies, diffusion enhancers are widely used to improve SEDDS mucopermeation. For example, PEGylated-type surfactants as diffusion enhancers in SEDDSs reduce SEDDS–mucus interactions by generating smaller SEDDS droplets with a negative to neutral zeta potential. Thus, supplementing SEDDS droplets with diffusion enhancers may enable them to glide more freely through mucus [5,6]. Furthermore, the positive transformation of mucopenetrating SEDDS droplets from a negative surface zeta potential ensures that reverse diffusion is avoided [7,8]. Optimizing a drug-loaded mucopenetrating SEDDS does not lead to sufficient mucopermeation [9], therefore, structural changes among the mucus resilient meshwork formed by cross-linked mucin fibers is required. This can be achieved, for instance, by spiking SEDDS droplets with a prototype mucolytic agent actively interacting with mucus. In this study, we demonstrate the role of SEDDSs in providing optimal mucopermeation of a drug-loaded model by combining these strategies. This was accomplished by nominating an optimal physicochemical modification in the model drug-loaded SEDDSs and supplying them with a mucolytic load of *N*-acetylcysteine (NAC) affecting mucus-mucin fibers. In addition to its suitability for SEDDS incorporation in this study, NAC as a small molecular weight thiolated molecule is a potential prototype mucolytic agent [10,11]. However, the high hydrophilicity of NAC (solubility = 200 mg/mL), attributed to its low log P of ~−0.66 [12,13], represents a major mucopermeation shortcoming. The predicted instant NAC release due to its low log P [13] results in a diminished mucolytic function of the penetrating SEDDS droplets meant to be near the underlying absorptive epithelium. An instant NAC release from mucoactive SEDDSs is thought to correspond to inadequate mucopermeation induced by the mucolytic destructive impact on the mucus gel layer. SEDDS droplets, however, can be modified to overcome mucopermeation shortcomings associated with NAC instant release. Generating SEDDSs with NAC sustained release is predicted to demonstrate a periodic release of low NAC bursts. Thus, allowing a gradual mucolytic function of the traversing SEDDSs, while penetrating mucus layers [14,15]. According to a previous study, loading drug/polymer ionic complexes in SEDDSs significantly demonstrated drug sustained release [13]. Therefore, NAC sustained release from mucoactive SEDDSs, in this study, was attained via ionic complexation with anionic-exchange resin-based polymers having diverse lipophilicity. Elucidations of mucolytic pathways used for SEDDSs are abundant in the present literature, and can be summarized as nanodroplets of a thiol substructure load permitting direct disulfide–exchange reactions, thereby, breaking the cross-linked mucus glycoproteins. Furthermore, nanodroplets with mucolytic enzymatic decoration structurally alter the glycoproteins-based mucin fibers via breaking peptide bonds [4,14]. Adequate permeation of the mucus is possible with SEDDSs possessing mucoactive properties by incorporating either: a thiol substructure load of sufficient lipophilicity (log P > 3) or a mucolytic enzymatic decoration. For instance, incorporating the mucolytic enzyme trypsin into SEDDSs requires lipidization via ion pairing or covalent bonding. Furthermore, it was assumed that SEDDS mucopermeation is significantly encouraged by incorporating free lipophilic thiol conjugates with a sustained release property [16,17,18,19]. Considering this assumption in the present study, mucoactive SEDDSs owing to different lipophilicity in their load of NAC polymer ionic complexes were investigated in vitro for improved mucolytic goals that would optimize their mucopermeation. In this study, mucoactive SEDDSs maintaining NAC sustained release were developed and their mucopermeation potential was assessed using rheology, mucodiffusion, and intestinal mucosal residence time.

## 2. Materials and Methods

### 2.1. Materials

Branched-polyethyleneimine (PEI, MW = 25 kDa), polyoxyethylene (20) sorbitan monooleate (Tween 80), 5,5′-dithiobis-(2-nitrobenzoic acid) (DTNB^2−^), *N*-acetylcysteine (NAC), macrogolglycerol ricinoleate (Kolliphor EL), fluorescein diacetate (FDA), sorbitan trioleate (Span 85), and dipropylene glycol were purchased from Sigma-Aldrich (Vienna, Austria). Evonik Röhm GmbH (Darmstadt, Germany) gifted the methacrylate-copolymers Eudragit^®^ E 100 (poly(butyl methacrylate-co-(2-demethylaminoethyl) methacrylate-co-methyl methacrylate), 1:2:1) and Eudragit^®^ RS 100 (trimethylammonioethyl methacrylate, methyl methacrylate, and ethyl acrylate, 0.1:2:1). 2-n-Octyl-1-dodecanol as an oil phase was purchased from Tokyo Chemical Industry Co., Ltd. (Tokyo, Japan). The remaining materials and solvents were of analytical grade and purchased from commercial sources.

### 2.2. Generation of NAC Ionic Complexes

NAC hydrophobic ionic complexes with different polymers, listed in Table 1, were generated. The NAC carboxylic group in charge-equimolar amount was loaded in ionic interactions with PEI and Eudragit E 100 (non-quaternary amine-based polymers) as well as Eudragit RS 100 (quaternary amine-based polymer), generating PEI–NAC, Eudragit E 100–NAC, and Eudragit RS 100–NAC complexes, respectively. Standard acetone solutions constituting 20 mg NAC in 2 mL tubes were generated in final volumes of 0.5 mL or 0.2 mL. In a respective manner, the same acetone volumes were utilized for the dissolution of PEI (5.1 mg) and Eudragit E 100 (30.7 mg), and then added dropwise to the aforesaid NAC solutions, respectively, being shaken at ambient temperature. On the one hand, the equimolar polymer-to-NAC ratio used above for generating PEI–NAC and Eudragit E 100–NAC complexes was 1:1, and another 2:1 ratio was utilized by increasing the amount of the polymer dissolved in acetone. On the other hand, methanolic solutions were utilized to generate the Eudragit RS 100–NAC complex in a polymer-to-NAC ratio of 1:4. Briefly, Eudragit RS 100 (54.1 mg) methanolic solution in 980 µL methanol was produced and mixed with 5 M NaOH pipetted as 20 µL. In a dropwise manner, a 100 µL volume fraction was added from a methanolic NAC stock solution (100 mg/mL) to the solution being shaken, achieving the above-desired polymer-to-NAC ratio. MiniSpin^®^ was used to centrifuge the precipitates for 5 min at 10,746× *g* (Eppendorf AG, Hamburg, Germany). Ellman’s buffer at 0.5 M and pH 8 comprising 0.05% DTNB^2−^ was used to dilute the supernatants and reference solutions omitting polymers. The dilutions were both transferred to a UV microplate reader to measure the absorbance determined at 450 nm. Absorbances indicating the presence of NAC were utilized in Equation (1) and the precipitation percentage was calculated. Acetone wash was followed for NAC precipitates represented by PEI and Eudragit E 100 complexes that were subsequently brought to immediate dryness. Moreover, precipitates of the Eudragit RS 100–NAC complex were lyophilized at −80 °C following demineralized water wash. Mucoactive SEDDSs were developed based on incorporating these complexes:

(1) $$\mathrm{Complexed}\;\mathrm{NAC}\;\left(\%\right)=\left(1-\frac{\mathrm{Supernatant}}{\mathrm{Reference}\;}\right)\times 100\%$$

**Figure 1:**
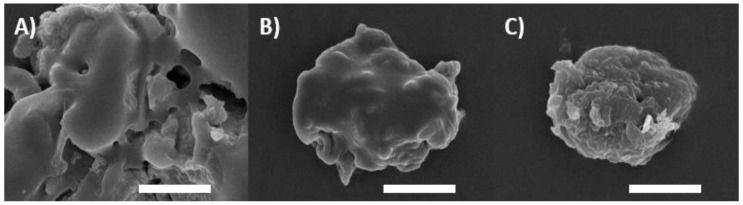
SEM images: (**A**–**C**) Characterizing NAC polymer ionic complexes with PEI, Eudragit E 100, and Eudragit RS 100, respectively.

Ionic complexes were characterized using scanning electron microscopic (SEM) imaging. Following the dissolution of complexes in methanol, 10 µL droplets of the solubilized complexes were placed on carbon tapes and incubated at 25 °C until dryness. Complex droplets adsorbed to carbon tape using Polaron SEM coating unit E5100 were coated with a 10 nm thick gold layer. Samples were placed in a JEOL JSM-6010 LV scanning electron microscope for imaging. The SEM images of the acquired NAC polymer ionic complexes are shown in Figure 1A–C.

### 2.3. Fourier Transform Infrared (FT-IR) Spectroscopy of NAC Ionic Complexes

Bruker ALPHA Fourier transform infrared spectroscopy (Billerica, MA, USA) was used to record IR spectra for NAC, polymers, and their complexes. Using a range of 4000–400 cm^−1^ wavenumber and a speed of 4 cm^−1^, solids were placed on the tip of a platinum attenuated total reflection instrument and scanned 32 times. 

### 2.4. Development of Mucoactive SEDDSs

The novel mucoactive SEDDSs in this study were developed by mixing various excipients with the obtained complexes described above. Firstly, the surfactants Tween 80, Kolliphor EL, and Span 85, the cosolvent dipropylene glycol, and the oil phase 2-n-octyl-1-dodecanol were blended at 20% (*v*/*v*) each, as adapted from previous works reporting SEDDSs [6,20]. Next, the SEDDS mixture was admixed to dissolve the pure complexes in the 2 mL tubes obtained above. Each tube contained 1, 1, and 0.5 mL volumes of mucolytic SEDDSs signifying NAC at about ~1.5%, ~1%, and ~0.5% (mass/vol.) from PEI, Eudragit E 100, and Eudragit RS 100 complexes, respectively. Pure complexes’ weights were identified from the mass difference of the tubes before and after preparation. Complexes’ initial dissolutions were begun with dipropylene glycol using vortex and up to 50 °C heating, and then left incubated overnight on a shaker at room temperature. A stock solution composing the above-specified ratios of the other excipients was added to dipropylene glycol solutions with mixing, and subsequently, three homogenous SEDDSs bearing different mucolytic NAC loads as polymeric complexes were developed. To characterize the SEDDSs, aliquots of 100 µL of SEDDS homogeneous preconcentrates were separately diluted in a release medium containing 0.05% DTNB^2−^ at pH 6.8 in 10 mL final volumes. Dilutions were placed on an Eppendorf Thermomixer (Hamburg, Germany) at 37 °C and 300 rpm shaking force. Afterward, 1 mL aliquots were taken at 0 h and 4 h and transferred to a particle sizer (Zetasizer Nano ZSP, Malvern Instruments Ltd., Worcestershire, UK) to measure the SEDDS droplet’s size and polydispersity index (PDI). SEDDS droplet’s zeta potential was measured after dilution in 10 mM phosphate buffer medium with pH 6.8. 

### 2.5. SEDDS Biological Safety 

Using the resazurin test, biocompatible SEDDS concentrations on the Caco-2 cell line were determined. Briefly, Caco-2 cells at 2.5 × 10^4^ cells per well in 24 well-containing plates were refreshed on an alternative daily basis with minimal essential medium (MEM) under optimal incubation conditions at 37 °C and 5% CO_2_. MEM replenishment was considered regularly until thick Caco-2 cell monolayers were reproduced. In 25 mM HEPES-buffered saline (HBS) at pH 7.4, SEDDSs containing the NAC polymer ionic complexes were diluted at 1:100 and 3:100. Positive and negative controls were HBS as a blank and 0.05% (mass/vol.) Triton-X^®^ 100 solutions, respectively. Under a light microscope, Caco-2 cell layers were confirmed, and thereafter, cells were washed thrice with preheated (37 °C) HBS. Caco-2 cells were incubated for 4 h with the positive and negative controls, as well as the tested SEDDS dilutions were added in triplicate at 500 µL/well. After that, each well was washed twice with prewarmed HBS, and 250 µL of 2.2 µM resazurin solution was added to each well. Following a 3 h incubation period at 37 °C and 5% CO_2_, aliquots of 100 µL were transferred to 96-well black microplates, and fluorescence was quantified with a microplate reader utilizing excitation (540 nm)/emission (590 nm). According to Equation (2), cell viability was defined as a function of fluorescence recovery based on viable cells’ abilities to produce the pinkish resorufin by reducing resazurin as compared with 100% fluorescence produced by the positive control:

(2) $$Viable\;Cells\;\left(\%\right)=\frac{\mathrm{Average}\;\mathrm{SEDDS}\;\mathrm{treatment}\;\mathrm{fluorescence}\;}{\mathrm{Average}\;\mathrm{positive}\;\mathrm{control}\;\mathrm{fluorescence}\;}\times 100$$

### 2.6. NAC Polymer Ionic Complexes Phase Partitioning 

The solubility test was performed in two phases: the lipophilic phase represented by n-octanol and SEDDS excipients, and the aqueous phase represented by deionized water and 0.05% DTNB^2−^-containing 0.1 M sodium phosphate buffer solution at pH 6.8. Increasing amounts of the NAC polymeric ionic complexes were completely dissolved in all phases. Free NAC dissolution was used as a control. All samples were kept for 24 h under 550 rpm shaking force at 37 °C using a thermomixer. All phases after producing clear solutions were separately aliquoted in Eppendorf tubes (2 mL) among which the n-octanol phase was lyophilized. Aliquots from all phases were diluted in sodium phosphate buffer (0.5 M at pH 8) containing 0.05% DTNB^2−^ at the same final volume considering predissolution of the n-octanol phase leftover in acetone that was subsequently evaporated. Following 4 h of additional shaking at 37 °C, the diluted samples were centrifuged, sub-aliquoted (100 µL), and their sub-aliquots were analyzed regarding absorbance at 450 nm via a microplate reader. The obtained absorbances of the dissolved NAC were substituted in Equations (3) and (4) to calculate the partition coefficients.

(3) $$Log\;D_{SEDDS/release\;medium}\;=\;log\left(\frac{dissoluted\;concentration\;in\;SEDDS}{dissoluted\;concentration\;in\;release\;medium}\right)$$

(4) $$Log\;P_{n-octanol/water}\;=\;log\left(\frac{dissoluted\;concentration\;in\;n-octanol}{dissoluted\;concentration\;in\;water}\right)$$

### 2.7. Ellman’s Assay for Released NAC Detection 

**Figure 2:**
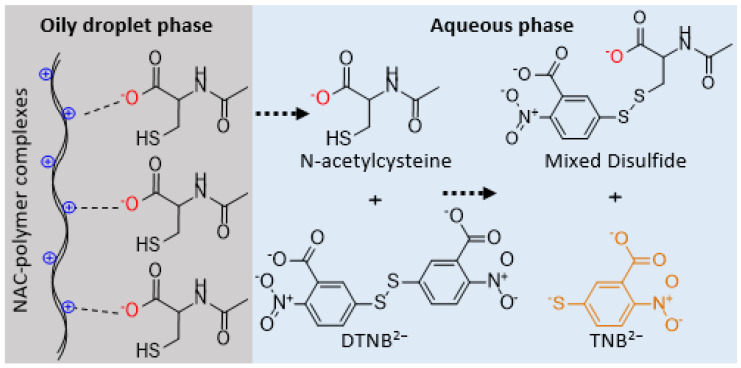
A diagram depicting the quantification approach of the released *N*-acetylcysteine from SEDDS oily droplets. TNB^2−^, 2-nitro-5-thiobenzoate dianion; DTNB^2−^, 5,5′-dithiobis(2-nitrobenzoic acid) dianion.

The thiol substructure of NAC was utilized in Ellman’s assay to feature its release from the SEDDSs [21]. For this aim, DTNB^2−^ or Ellman’s reagent was dissolved at a concentration of 0.05% in 0.1 M sodium phosphate Ellman’s buffer solution at pH 6.8 was employed as a thiol detector upon NAC release from SEDDSs. Ellman’s reagent (5,5′-dithiobis-(2-nitrobenzoic acid)) undergoes a disulfide–exchange reaction with thiol substructures to form 2-nitro-5-thiobenzoate dianion (TNB^2−^), a spectrophotometrically sensitive product that quantitatively measures how much sulfhydryl groups reacted in a solution [22]. Furthermore, the considerable hydrophilicity of DTNB^2−^ in Ellman’s buffer prevents its reverse diffusion into SEDDS droplets, and thus, permits the disulfide exchange with the released NAC only. As a result, this quantification method provides a real-time selective disulfide exchange process, signifying its appropriateness for NAC detection. Figure 2 shows the full process in schematic form. The mucolytic SEDDSs containing different complexes were emulsified in the release medium at a 1:100 ratio using multiple Eppendorf tubes (2 mL) per formulation for a duration of 2 h using a 70 rpm plate shaker at 37 °C. At 20 min intervals, 100 µL aliquots were taken, in triplicate, and transferred to 96-well microplates. A microplate reader (Tecan Spark^®^, Tecan Trading AG, Zurich, Switzerland) was used to measure the release of NAC from the SEDDSs at each time point at a wavelength of 450 nm. The reference positive control utilized methanolic NAC polymer ionic complex solutions diluted in the release medium. The uprise in the absorbance from SEDDS dilutions detected at each time-point was substituted in Equation (5) to figure out the percentage of the released NAC from SEDDSs in correlation to the reference 100% control. To predict NAC release, the Nernstsches equation (Equation (6)) [23] was used to estimate the NAC percentage that would remain in SEDDS droplets without release by identifying the ratio of D_SEDDS/release medium_ found in Equaiton (3) as follows:

(5) $$\mathrm{NAC}\;\mathrm{in}\;\mathrm{release}\;\mathrm{medium}\;\left(\%\right)=\frac{absorbance\;read\;at\;a\;given\;time-blank\;absorbance}{reference\;absorbance}\times 100$$

(6) $$SEDDS\;remaining\;solute\;\left(\%\right)=\frac{100\%}{1+\frac{Volume\;of\;release\;medium}{Volume\;of\;SEDDS\times DSEDDS/release\;medium}}$$

### 2.8. Calibration Curve 

NAC was dissolved using different dilutions in the release medium at concentrations ranging from 0.0025 to 0.02%, to address the above released NAC from SEDDSs. A spectroscopic analysis to read absorption at 450 nm was performed on all dilutions that were aliquoted in triplicate using a microplate reader.

### 2.9. Mucus Viscoelastic Alterations 

The rheological changes on intestinal porcine mucus influenced by the released mucolytic NAC from SEDDSs were in vitro analyzed via a rheometer (Haake Mars Rheometer, 379-0200, Thermo Electron GmBH, Karlsruhe, Germany). Following a previous protocol, mucus dynamic viscosity (η), elastic modulus (G′), and viscous modulus (G″) were investigated [24]. Collection and purification of mucus from pig intestines were performed according to a previous protocol [5]. Fresh pig intestines were supplied and kept on ice from a neighboring butcher. Dissected intestinal segments containing chyme were discarded. After the bulky waste was removed, intestinal segments of approximately 10 cm in length were longitudinally incised to scrape and collect the mucus in a homogeneous manner. A suspension of 200 mg/mL of the collected mucus in 0.1 M sodium chloride was gently swirled for an hour and subsequently centrifuged to yield purified and clear pellets that were maintained at −20 °C until needed. At a ratio of 3:10, SEDDSs containing the NAC polymeric complexes were diluted in a phosphate buffer medium (0.1 mM) with pH adjusted at 6.8. At 3:10 dilution in buffer, a blank SEDDS was treated as the negative control. All of the dilutions at a volume of 1 mL were mixed 1:1 (vol./mass) with 1 g of the purified mucus and incubated at 37 °C for 2 h in an airtight environment. For each mucus-formulation mixture, mucus viscoelastic changes were monitored utilizing five samples at 30 min time intervals for 120 min. As a positive control, the viscoelastic changes caused by the tested formulations were figured out within a calibration of 0–0.857% free NAC in buffer combined with the blank SEDDS and the mucus under the incubation conditions as indicated above, to produce 0, 0.037, 0.07, 0.15, and 0.30% final NAC concentrations. Using a plate-plate combination rheometer, the setup of viscoelastic measurements of all samples included a shear stress rate ranging between 0.1 and 2 Pa, a frequency of 1 Hz at a temperature of 37 ± 1 °C, and a chosen gap of about 0.5 mm between two plates. Under these conditions, HAAKE RheoWin 3 software was used to find a linear area indicating the optimum measurement of samples’ viscoelastic changes. 

### 2.10. SEDDS Mucopermeation Studies

#### 2.10.1. Transwell Mucodiffusion Model

The static transwell mucodiffusion model was used in vitro to evaluate mucoactive SEDDS diffusion through the intestinal mucus. Quantification of SEDDSs permeating the mucus was aided with a fluorescence tracer labeling the permeated SEDDSs. For this aim, the mucoactive and blank SEDDSs were labeled with the model drug FDA using acetone. An acetone solution of FDA at a concentration of 10 mg/mL was formed and pipetted at a volume of 100 µL into each tube containing 500 µL of SEDDSs. In reference to Section 2.4, 0.5 mL of SEDDSs comprising the NAC polymer ionic complexes were extracted from the prepared original volumes. The reconstituted acetone with SEDDSs was then evaporated for 60 min at 800 rpm and 37 °C in a thermomixer and a final FDA load in SEDDSs of 0.2% was obtained. For mucus permeation studies, 24-well transwell plates with inserts having a surface of 33.6 mm^2^ and pore size of 3 µm (Greiner Bio-One, Kremsmunster, Austria) were used and these inserts were covered with 50 mg of intestinal mucus. The upper donor compartment of inserts attaching a tightly sealed filtering membrane (pore size = 3 µm) at the bottom was gently filled with 250 µL of pH 6.8 phosphate buffer (0.1 M) containing 1:100 dilutions of the tested SEDDSs. Conversely, the acceptor compartment directly underneath the filtering membrane held 500 µL of the same blank buffer. Wells comprising the positive and negative controls had donor compartments filled with a blank SEDDS and an FDA-labeled SEDDS diluted in buffer, respectively, in absence of the mucus. The test and control samples were incubated for 4 h on a plate shaker with a gentle 75 rpm shaking force at 37 °C and experimented in triplicate. During this, 100 µL aliquots on an hourly basis were drawn from all of the acceptor compartments with fresh buffer replacement. Aliquots were transferred to 96-well plates and mixed with 10 µL of 5 M NaOH to ensure FDA hydrolysis and fluorescein release following additional incubation for 30 min. The cumulative fluorescence detected instrumentally at 480 nm/520 nm (excitation/emission) using a microplate reader was used to calculate the percentage of the permeated SEDDSs.

#### 2.10.2. Scanning Electron Microscopic Imaging of Permeated SEDDSs

The permeated mucoactive SEDDS droplets from the transwell model above were confirmed using SEM images following the method mentioned earlier (Section 2.2). Aliquots of 400 µL were taken into 1 mL tubes at the end of transwell diffusion from the acceptor chambers that were incubated for the full duration without sampling. Aliquots were freeze-dried and cumulating SEDDSs were separated from buffer ionic strength using acetone. Acetone was evaporated and the remaining SEDDSs were dispersed in 100 µL deionized water. Droplets of the dispersed SEDDSs were placed on carbon tapes and incubated at 25 °C until dryness. The SEDDS droplets adsorbed to the carbon tape and were coated with a 10 nm thick gold layer and placed on a JEOL JSM-6010 LV scanning electron microscope at 15,000× magnification for SEM imaging performed under high vacuum at 15 kV acceleration voltage.

#### 2.10.3. SEDDS Residence Time on Intestinal Mucosa 

The ability of SEDDSs to remain resident on intestinal mucosa was measured quantitatively in vitro over a specific time using a previously developed method [25]. Plastic grooves were employed to fit porcine intestinal slices of 2 × 3 cm dimensions that were dissected from a fresh pig intestine. The FDA-labeled SEDDS preconcentrates mentioned above were individually applied at 100 µL to the dissected intestinal mucosal slices. The slices were kept in a flat position for 10 min to allow them to disseminate evenly. Then, the slices were incubated at 37 °C for 4 h and angled at a 45° angle with the SEDDS dispersions on the upper side. A pressure-driven pump was used to flush the intestinal mucosa at a flow rate of 1 mL/min, allowing buffer at 0.1 M (pH 6.8) to pass through. Slices containing FDA-labeled SEDDSs but not flushed served as a positive control, whereas slices containing unlabeled SEDDSs served as a negative control. An additional negative control considered the use of slices devoid of SEDDSs. Slices were soaked in 20 mL of 5 mM NaOH, vortexed following 4 h of incubation, and incubated for an additional 45 min with stirring to hydrolyze FDA. At excitation/emission wavelengths of 480 nm/520 nm, fluorescence intensity produced by the tested SEDDS was compared to the positive and negative controls to measure the amount of SEDDSs that remained resident on the intestinal mucosa. 

### 2.11. Statistical Analysis

GraphPad Prism V. 7.05 was used for statistical analysis. Two-way ANOVA described experimental significances using the 95% confidence interval (*p*-value ≤ 0.05). Significant differences between data points were classified as * *p* ≤ 0.05 (significant), ** *p* ≤ 0.01 (very significant), and *** *p* ≤ 0.001 (highly significant). The linear regression square (r2) describing the line graph of different NAC concentrations in Section 2.8 was found to be 0.991 ± 0.003. The indicated data are expressed as means of at least three experiments ± standard deviation.

## 3. Results and Discussion

### 3.1. NAC Polymer Ionic Complexes

Corresponding to the polymer solutions added drop wisely, NAC dissolved in different solvents formed the target hydrophobic ionic complexes indicated in Table 1 by showing immediate whitish precipitates. As compared with the 2:1 ratio, PEI at a 1:1 ratio to NAC showed around 4.2% more precipitated NAC with a final 74% of the precipitated NAC in the PEI–NAC complex. Conversely, Eudragit E 100 produced 55.9% precipitated NAC, which sharply decreased as the polymer-to-NAC ratio was increased. As a result, for PEI–NAC and Eudragit E 100–NAC complexes, a polymer-to-NAC ratio of 1:1 showing a higher NAC precipitation percentage (Table 2) was selected for the development of mucoactive SEDDSs. Furthermore, Eudragit RS 100 in the Eudragit RS 100–NAC complex should be demanded in a 4-fold higher amount to fully interact with the utilized NAC amount. However, a 1:4 Eudragit RS 100-to-NAC ratio was used instead, since the ratio at 1:1 required a significantly larger amount of Eudragit RS 100 (~216.4 mg). Therefore, the amount of Eudragit RS 100 in this complex and its final concentration in SEDDSs were reduced as possible with the used ratio. In addition, a fully saturated polymer in the Eudragit RS 100–NAC complex had precipitation of 26.2% from the utilized NAC amount, according to the ratio used. This complexation behavior is congruent, to some extent, with the concept that ion-pairing equilibria are better obtained between ionic counterparts being used in equivalent charge ratios [18]. The approach for creating the anticipated NAC complexes with the polymers significantly relied on lowering the dielectric constant of the used solvents [26,27]. The non-quaternary amino groups in PEI and Eudragit E 100 require a step of proton shift from NAC to generate a cationic ammonium intermediate that spontaneously pairs with NAC carboxylate. Acetone as a medium for ion pairing offers a dual advantage in that its low dielectric constant of 22 is more favorable for ion pairing and its aprotic nature enables salt conversion of the polymer’s non-quaternary amino groups by aiding proton transfer, as previously reported [28]. In contrast, the readily activated charge of the permanent quaternary ammonium group of Eudragit RS 100 requires direct electrostatic interaction with the NAC carboxylate, which has a pKa of 3.24 [12]. Therefore, the use of NaOH possibly led to deprotonation of the NAC carboxyl group in the methanolic ion-pairing medium, which has a relatively low dielectric constant, which also supported the required ionic interaction [29]. Incorporating hydrophobic ionic complexes with varying NAC concentrations in SEDDSs is anticipated to exhibit different mucopermeation qualities provided that a gradient of mucolytic activity can be observed.

### 3.2. FT-IR Spectroscopy of Hydrophobic Ionic Complexes 

**Figure 3:**
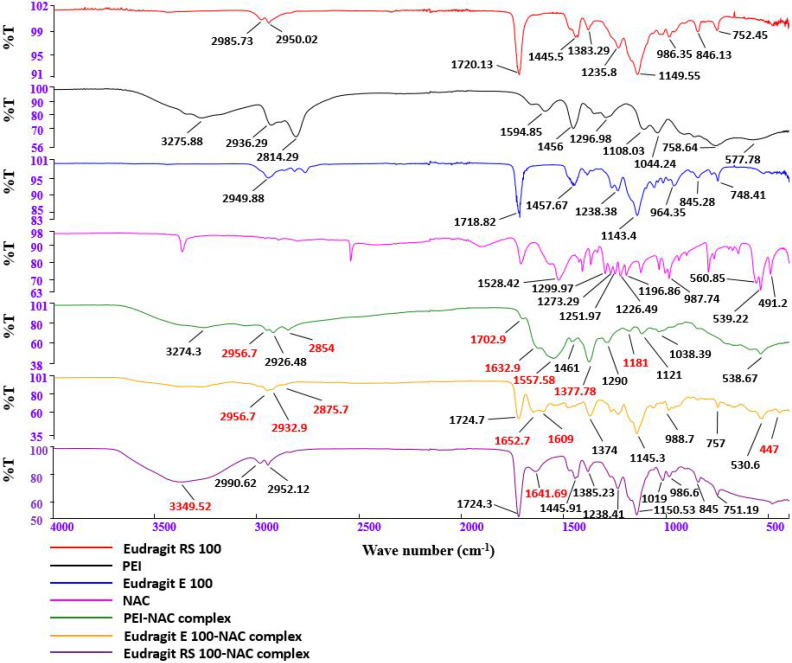
FT-IR spectroscopy of *N*-acetylcysteine, polymers (PEI, Eudragit E 100, and Eudragit RS 100), and NAC polymer ionic complexes.

The IR spectra of free NAC, polymers, and NAC polymer ionic complexes are illustrated in Figure 3. The existence of distinctive peaks related to cationic activation of polymeric primary, secondary, and tertiary amino groups needed for ionic bond formation is the most conspicuous aspect of the IR spectra. The PEI–NAC complex showed a succession of weak bands in the range of 2853.9–2956.7 cm^−1^, including the complex’s distinctive wider PEI band coverage at 3274.5 cm^−1^ showing a wide range untill ~2000 cm^−1^. These peaks are very similar to previously reported peaks in the 3200–2000 cm^−1^ range, which are typical of primary amine-salt [27]. According to the previously reported ephedrine sulfate band at 2860 cm^−1^ [28], the stretch at 2853.9 cm^−1^ might be given to the PEI secondary amine salt. The PEI–NAC complex’s bands between 1500 and 1700 cm^−1^ may potentially be responsible for PEI secondary amine salt formation [28]. In addition, the Eudragit E 100’s tertiary amino groups evidenced by the novel absorption bands at 2875.7 and 2932.9 cm^−1^, may entitle salt transformation [27]. Finally, the Eudragit RS 100–NAC complex showed novel spectral features at 1641.69 and 3349.52 cm^−1^.

### 3.3. Mucoactive SEDDS Characterization

**Figure 4:**
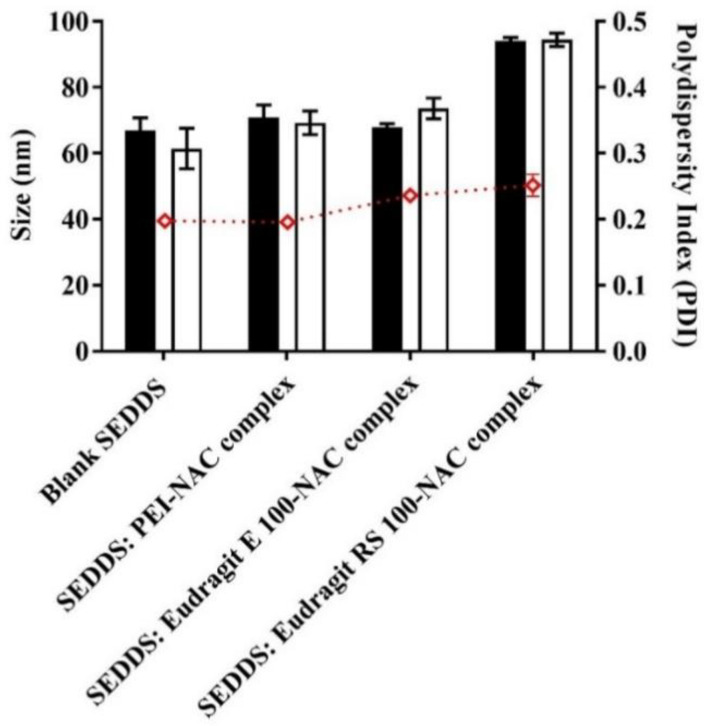
Average droplet size at 0 h (black bars) and 4 h (white bars) and the polydispersity index (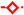) of different SEDDS droplet types classified as a blank SEDDS, and SEDDSs loading three different NAC ionic complexes with PEI, Eudragit E, and Eudragit RS, following 1:100 dilution in release medium at 37 °C. The values given are the means ± standard deviations (*n* ≥ 3).

**Figure 5:**
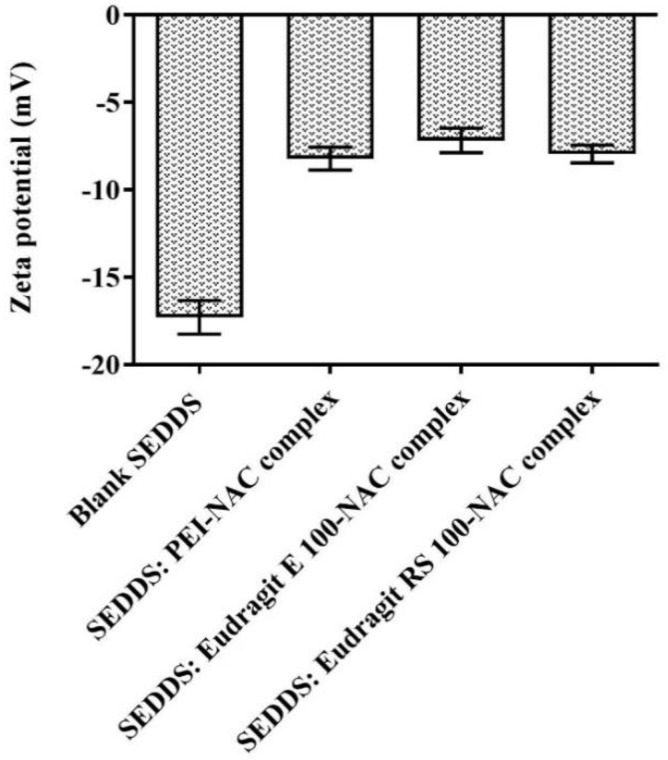
The average values of the zeta potential of 1:100 diluted SEDDSs showing a blank SEDDS and SEDDSs of polymeric NAC ionic complexes determined in phosphate buffer with 10 mM ionic strength at pH 6.8 at 37 °C. The values given are the means ± standard deviations (*n* ≥ 3).

SEDDS dispersion in the diluting release medium was achieved in a matter of seconds after gentle shaking using a thermomixer. The presence of DTNB^2−^ in the aqueous release medium yielded the disulfide exchange end product of the reacting free NAC upon its instant release from the SEDDSs. For verification, the resultant discolored solution showing full fluorescence recovery was evaluated by measuring the absorbance. During a period of 4 h size measurement, according to Figure 4, all of the SEDDSs showed an average droplet size of 75 ± 12 nm. Furthermore, all of the size measurements indicated SEDDS droplets’ stability and uniform distribution by preserving < 0.3 PDI. After ≥24 h, the size data preserved similar stability (PDI < 0.3) and showed an overall SEDDS average droplet size of 75.05 ± 13 nm elucidated as: 65.2 ± 0.6 nm, 69.7 ± 4.9 nm, 70.9 ± 2 nm, and 94.2 ± 1.1 nm individual average droplet size for the blank SEDDS, and the SEDDSs loading PEI, Eudragit E 100, and Eudragit RS 100 complexes, respectively. Except for the SEDDSs loading Eudragit RS 100–NAC, which showed an increase in droplet size by 1.4-fold, the other complex-loaded SEDDSs were similar in size to the blank SEDDS. A previous evaluation reported similar sized increases of SEDDS droplets exhibited as 1.8- and 1.5-fold following the incorporation of octylamine at 5% and 1-decyl-3-methylimidazolium chloride at 1%, respectively, as stated by Lam et al. [30]. SEDDSs with a droplet size of less than 100 nm can pass through the mucus gel layer with considerable ease [6,31]. According to Friedl et al., the inverse association between the size of SEDDS droplets and their diffusion capacity through the mucus layer resulted in about 70% of a model drug penetrating the mucus as a load of SEDDS droplets exhibiting a small size of 12 nm. In contrast, SEDDSs that dubbed a relatively large diameter of 455 nm showed only 8% model drug permeating the mucus [5]. A preset mucopore size of 200 nm accounting for >90% of the mucopores was utilized in the methods for gathering and purifying porcine mucus, allowing diffused SEDDSs to penetrate efficiently even with a SEDDS droplet size exceeding 100 nm [32]. Furthermore, the NAC’s mucolytic activity was assumed to allow the generated SEDDSs to circumvent the mucopore size barrier [15]. As compared with the blank SEDDS with a droplet size of 50 nm showing 10% mucopermeation of a loaded model drug based on a previous report, SEDDSs exhibiting significantly larger droplets sizes of 180–312 nm but still anchoring a mucolytic load of papain-, trypsin-, and bromelain-conjugate produced roughly 25–45% higher mucopermeation of the loaded model drug [19]. Additionally, supplementing the PEGylated-type surfactants Kolliphor EL and Tween 80 in the final SEDDS composition influenced the desired deformative and flexible shape of the SEDDS droplets generating a slippery SEDDS droplet surface which facilitated their diffusion across the mucus gel layer [5,6]. Furthermore, SEDDS droplets’ surface charges regulated mostly by zeta potential preferably allow neutral or negatively charged droplets to permeate the mucus layer in a more efficient manner [7,33]. Mucus substructures having a great abundance of sialic acid, sulfonic acid, and phosphate groups as negatively charged moieties tend to interact with SEDDS droplets showing a significant positive zeta potential transformation. In turn, this would cause serious entrapment and limited penetration of the mucus [34,35]. The zeta potential values of the SEDDSs, according to Figure 5, were obtained following multiple dilutions in phosphate buffer at pH 6.8 and 10 mM ionic strength. The blank SEDDS showing −17.31 mV average zeta potential shifted to a −7.72 mV higher average when the polymeric NAC complexes were incorporated. The persistence of the negative charge on the SEDDS surface is still consistent with the requirements for efficient mucopermeation even with the subsequent incorporation of the complexes. Therefore, the SEDDSs of the NAC polymeric ionic complex load, while preserving the desired negative zeta potential values, did not create a gap with the blank SEDDS under the same pH settings in terms of diffusion through the mucus layer harboring negatively charged proteoglycans [19]. In this study, the physicochemical properties of the developed SEDDSs were integrated. Therefore, the presence of a mucolytic load in these SEDDSs would likely be another key factor to optimize their mucopermeation. 

### 3.4. SEDDS Safety on Caco-2 Cells 

**Figure 6:**
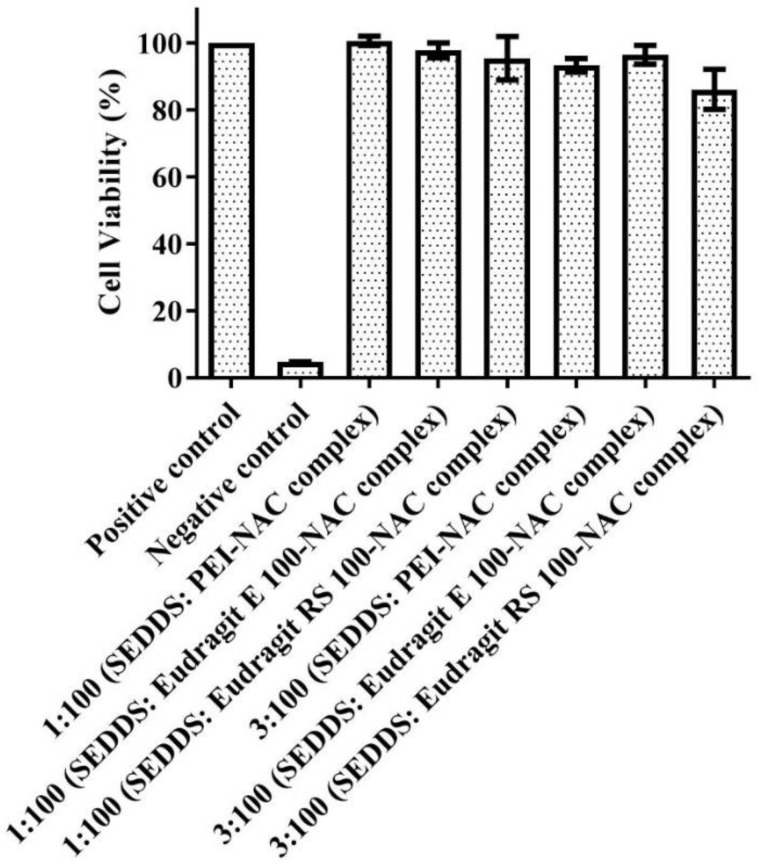
Caco-2 cell viability percentages in the resazurin test following treatment of 1:100 and 3:100 dilutions of the SEDDSs bearing various NAC complex loads in HBS (pH 7.4) after a 4 h incubation period at 37 °C. The data iare provided as the mean ± standard deviation of three experiments.

At 1:100 and 3:100 dilutions in the cell culture medium, all SEDDS preparations were deemed to be safe throughout incubation for 4 h. According to Figure 6, the range of cell viability between 85 and 100%, indicating optimal resazurin transformation to resorufin by Caco-2 cell, proved to be a high safety limit of these dilutions. The studied Eudragit family of polymers exhibited a high safety limit, despite the fact they possessed a large abundance of positively charged amino substructures with the assumption of incurring potential toxicity on cells whose cell membranes were at the resting negative potential [36]. Using lipid-based nanostructured carriers formed from Eudragit RS 100, Zhang et al. found that genistein had no deleterious effect on human corneal epithelial cells at a concentration up to 100 µg/mL [37]. In addition, we previously reported >80% viability of Caco-2 cells using the same SEDDS dilutions as the current study by using 1.8% Eudragit RS 100 and 1.07% Eudragit E 100 in SEDDS formulations [13]. On the contrary, PEI possessing a relatively greater abundance of the positively charged amino groups as compared with any polymer is considered potentially cytotoxic. Depending on the targeted cellular components, PEI-mediated cytotoxicity can manifest as direct interaction with cell membranes causing rapid necrosis or slow damage to mitochondria causing delayed apoptosis [38]. The integrated PEI complex comprising 0.015% PEI had no toxic potential in the SEDDS dilutions utilized in the experiments. This agreed with previously reported PEI toxicity data by Edet et al. for the same PEI on HeLa cells. In their evaluation, they found a PEI cytotoxic limit starting at dilutions of 2 mg/mL during a 72 h incubation period, and that employing lower concentrations in PEI dilutions ensured 100% cell viability [39]. Furthermore, the adopted change involving ion pairs and subsequent SEDDS integration might explain the lower PEI toxicity [40,41].

### 3.5. Mucoactive SEDDS Release Studies

**Figure 7:**
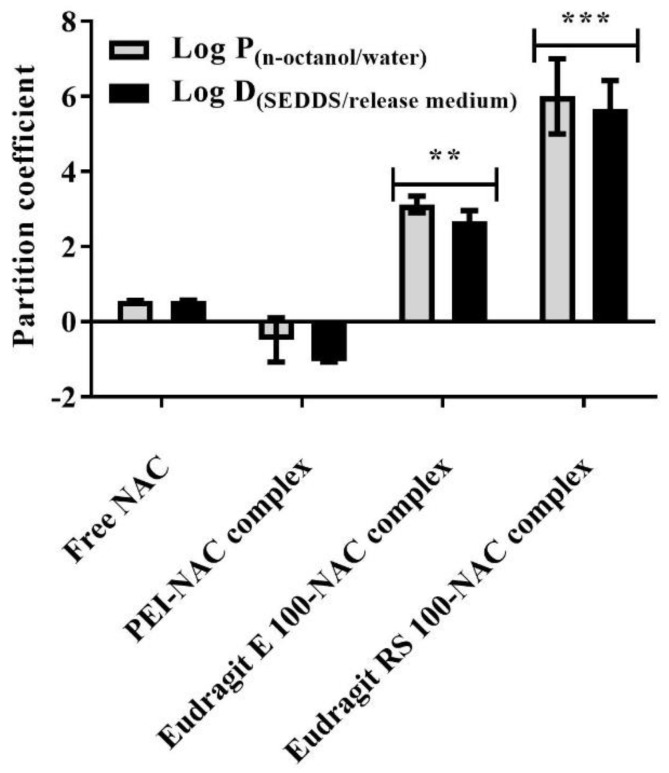
The partition coefficients log P_n-octanol/water_ and log D_SEDDS/release medium_ were determined at 37 °C in relation to free NAC as well as NAC complexes with PEI, Eudragit E 100, and Eudragit RS 100 by measuring their solubility from the diluted organic phases of n-octanol and SEDDSs and aqueous phases of demineralized water and release medium in 0.05% DTNB^2−^ containing 0.5 M Ellman’s buffer (pH 8) using 450 nm absorption wavelength. In comparison to free NAC, *** *p* ≤ 0.001 and ** *p* ≤ 0.01 were found. The values given are the means ± standard deviations (*n* ≥ 3).

**Figure 8:**
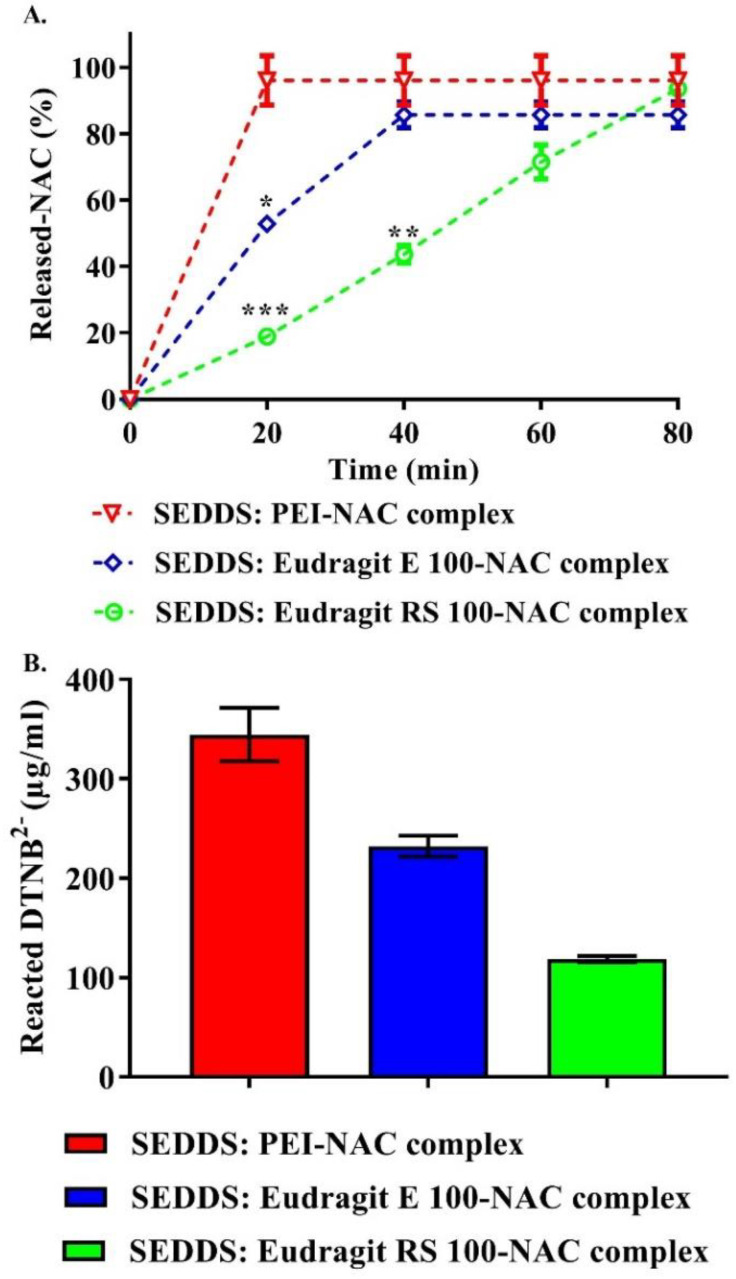
(**A**) NAC release profiles of the 1:100 diluted SEDDS complexes in a release medium of phosphate buffer (pH 6.8) comprising 0.05% DTNB^2−^ at 37 °C. The released NAC was measured using 450 nm absorption wavelength at 20 min intervals throughout an 80-min period; (**B**) at the end of the release, cumulative DTNB^2−^ concentration (µg/mL) that formed disulfide heterodimer by reacting with the released NAC was calculated in terms of molar equivalence to the quantified thiol substructure from NAC upon its release from SEDDS complexes. Eudragit E 100 and RS 100 showed a significantly slower NAC release rate from SEDDS than PEI (*** *p* ≤ 0.001, ** *p* ≤ 0.01, and * *p* ≤ 0.05). The means ± standard deviations (*n* ≥ 3) are provided.

The partition coefficient (log D) characterizing in situ drug release is primarily the most reliable model describing in vitro release kinetics from SEDDSs [23]. However, different physicochemical features of SEDDS droplets, such as nano-range diameter, surface zeta potential, and hydrophilic–lipophilic balance (HLB) could be secondary factors that influence drug release rate. For characterizing NAC release kinetics in this study, log D entitles that NAC, depending on its diffusion coefficient, has to overcome the barrier accounted between SEDDS droplets and the surrounding medium [23,42]. In reference to Equaiton (6) above, on the one hand, the log D values (highlighted between −1.5 and 0.5), according to Figure 7, predict >90% of NAC as free form or complexed with PEI to be released almost instantly from SEDDSs. The Eudragit E 100–NAC complex, on the other hand, with a log D of about ~2.6, suggests nearly ~20% immediate NAC release, whereas the Eudragit RS 100–NAC complex had a log D value up to ~6, indicating no immediate release. Since these two complexes featured non-releasing Eudragit polymers (log D > 5), a rate-limiting step elucidated as slow NAC dissociation and partitioning from the SEDDS organic phase to the aqueous release medium, therefore, was required to show NAC sustained release. Furthermore, complex solubility preferences described in log P showed a favorable partitioning of Eudragit E 100–/RS 100–NAC complexes in the n-octanol phase, whereas free NAC, as well as the PEI–NAC complex, showed a greater tendency toward the aqueous phase presented as demineralized water. Log P, in this study, was utilized as a useful tool to further aid in predicting the NAC release behavior from the produced SEDDS complexes. The illustrated release profiles in Figure 8A were compatible with the log D values predicting various rates at which NAC could be presented to the release medium from different SEDDS complexes. All of the SEDDS complexes released an overall average of 91.8 ± 5.4% NAC in the release medium. The PEI complex almost released NAC immediately within a few seconds from SEDDSs, whereas significantly slower release rates of NAC were contributed by the SEDDSs anchoring Eudragit RS 100 and E 100 complexes that released NAC in ≤80 min (*p* ≤ 0.001) and ≤40 min (*p* ≤ 0.05), respectively. In addition to the aforementioned sufficient lipophilic property of Eudragit E 100 and RS 100, these two polymers as per molecular unit possess a large molecular weight with around 188 and 10 amino cationic binding sites, respectively. Thus, they successfully trapped NAC in the SEDDSs through ionic bonding that significantly enhanced its lipophilicity and decreased its release rate. Eudragit RS 100’s permanent cationic ammonium substructures, however, generated stronger ionic bonds and provided more sustained NAC release than Eudragit E 100’s pH-dependent tertiary amine [43]. Nazir et al. also reported a significant sustained release of bovine serum albumin (BSA) in a physiological medium from SEDDSs, consequent to loading it in an ion-pair form with the anionic pamoic acid contributing to a considerable lipophilic enhancement of this therapeutic peptide in SEDDSs. The SEDDSs and release medium also established a slowly releasing BSA equilibrium in their experiment, which was found to be consistent with the ion pair’s log D [42].

Designing SEDDSs that regularly emit numerous low mucolytic bursts of a loaded mucolytic structure is a successful technique for SEDDS optimal mucodiffusion [14]. In addition to the evident mucolytic role of NAC, modifying a NAC slowly releasing SEDDSs can help them to permeate the mucus while maintaining their suitability for efficiently diffusing a multi-layered intestinal mucus. The researched mucoactive SEDDSs, to a considerable part, simulate the effect of specific bacteria migrating through the mucus by well-controlled localized production of mucolytic chemicals, impacting the surrounding mucus without entirely distorting it. The depicted NAC release patterns in Figure 8A were adequate for this purpose by showing a sustained release of NAC from the stabilized NAC polymeric ionic complexes into the formed SEDDS droplets. The DTNB^2−^-preserving activity induced an instantaneous disulfide–exchange reaction at pH > 6, allowing for the monitoring of time-dependent NAC release [21,44]. Figure 8B shows the calculated amount of DTNB^2−^ that formed a disulfide heterodimer with NAC subsequent to its entire release from SEDDS complexes. This was obtained by comparing the molar equivalence of DTNB^2−^ required to react with the quantified thiol substructure from NAC upon its presence in release medium. NAC that released nearly in full amount, highlighted about ~344.6, 232.2, and 118.8 µg/mL of reacted DTNB^2−^ with respect to NAC that released from PEI’s, Eudragit E 100’s, and Eudragit RS 100’s SEDDS complexes, respectively. Only by releasing NAC with a sulfhydryl group having a pKa of 9.8 [12] can a new disulfide heterodimer of DTNB^2−^ be formed [21]. Based on the obtained NAC release profiles, NAC that was released from SEDDSs at optimum level effectively underwent disulfide–exchange reaction with DTNB^2−^ and caused considerable yellow staining in the release medium. Provided that optimum NAC reactivity was attained from the release studies, the likewise disulfide–exchange interactions between the released NAC and mucus-cysteine subdomains could also predict significance.

### 3.6. Viscoelasticity Studies

**Figure 9:**
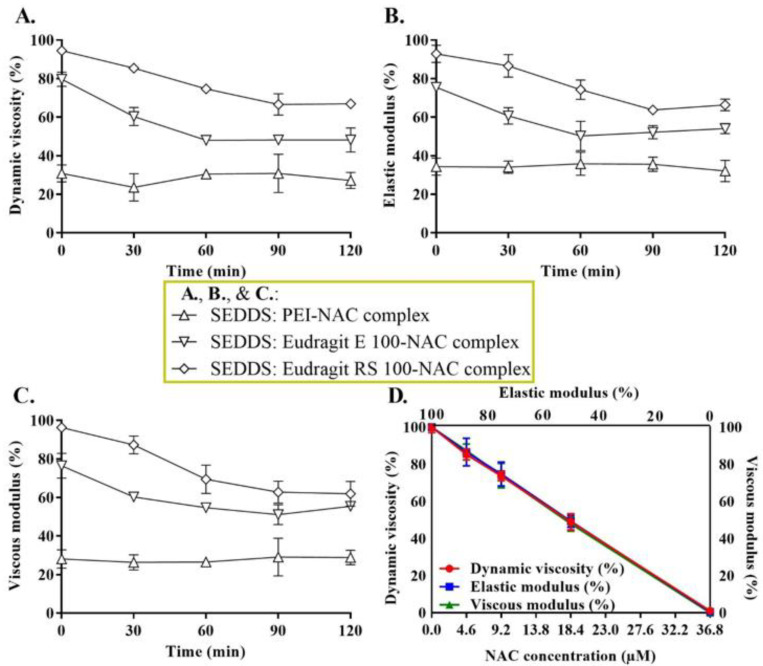
Viscoelastic changes over 2 h at 30-min intervals concerning dynamic viscosity (**A**), elastic modulus (**B**), and viscous modulus (**C**), influenced by different SEDDS complexes with 0.5–1.5% NAC loading, measured at 37 °C. The mucoactive SEDDSs in a ratio of 3:10 in 0.1 M phosphate buffer (pH 6.8) were admixed at 1:1 (vol./mass) with purified mucus. The associated viscoelastic changes of various NAC concentrations from mucolytic SEDDSs were addressed within the calibration curves (**D**) showing a gradient of changes in viscoelastic parameters affected by various NAC concentrations in the range of 0–36.8 µM in the same buffer volume with the SEDDS added as a blank in the same weight of mucus described above. The calibration curves utilizing various NAC ratios or omitting NAC were treated as the positive or negative controls, respectively, and considered likewise incubation conditions and time as the test samples. Indicated values are the means ± standard deviations (*n* ≥ 3).

Mucoactive SEDDS-influenced changes in mucus dynamic viscosity, viscous modulus, and elastic modulus were recorded using a well-established linear viscoelastic area, as mentioned above. A concentration-dependent calibration of various NAC concentrations ranging from 0 to 36.8 µM was utilized in measuring mucus viscoelastic changes, as shown in Figure 9D. In this calibration, the absence of NAC within the mucus mixture represented the negative control, whereas the positive control was shown as gradients of mucus viscoelastic changes with the increasing NAC-mucus content. The time-dependent impact of mucoactive SEDDS complexes bearing approximately ~0.5–1.5% NAC on mucus rheological parameters by the end of the test, as demonstrated in Figure 9A–C, seemed to be appropriately embedded within the range of NAC concentrations used for calibration. In this calibration (Figure 9D), the negative control was also included; mucus combined with buffer containing no NAC, but the blank SEDDS exhibiting no effect on mucus rheological characteristics. As compared with the blank SEDDS, the mucoactive SEDDSs demonstrated a significant drop (by approximately ~33–73%) in all rheological parameters in vitro, as illustrated in Figure 9A–C. Furthermore, all of these rheological changes were spotted in the calibration curves (Figure 9D) representing viscoelastic changes against different NAC concentrations mixed with mucus as the positive control. In finding a way to develop our calibrations, reported different mucus viscoelastic changes induced by mixing porcine intestinal mucus with several thiol conjugates by Rohrer et al. were adapted [16]. They also observed that mucus viscoelastic changes were similar when a thiol conjugate-spiked SEDDS was compared to a positive control of an equimolar dithiothreitol (DTT) integrated into a SEDDS. Furthermore, sufficient hydration of our samples (for testing and calibration) was achieved by adding 35% of the pH 6.8 phosphate buffer in the final homogenous mixture of mucus and SEDDSs in order to facilitate the desired disulfide–exchange reaction [15]. This enabled a time-dependent observation of mucus rheological changes that coincided with the slowly released mucolytic bursts of NAC undergoing disulfide exchange with mucin fibers’ cysteine subdomains under the same pH settings [14], just as it did with the previously mentioned NAC–DTNB^2−^ disulfide reaction (Section 3.5). The pH variability of the aqueous medium sinking the multi-layered mucus covering the small intestinal mucosa has a tremendous influence on the reactivity of thiol-bearing structures. The pH of the luminal side medium of the intestinal mucus that begins at pH 5 increases gradually to pH 7.4 towards the epithelial side and allows increased thiol reactivity of the permeating thiolated molecules [45,46,47]. Therefore, the choice of pH 6.8 simulation medium for forming the mucus mixture with mucoactive SEDDSs was appropriate to our in vitro rheological findings and could permit extrapolation to further in vivo investigations. To enhance mucopermeation to the largest extent in this study, the progressively penetrating SEDDS droplets have to reach deeper mucus layers where thiol reactivity is substantially improved due to the increased pH [14]. Therefore, the observed release pattern emphasizing the role of Eudragit E 100– and RS 100–NAC complexes in prolonging NAC release from SEDDSs suggests an in vivo benefit over other SEDDSs showing an immediate emptying of their mucolytic NAC load. Moreover, SEDDS droplets formed by flexible and deforming excipients were expected to be partially suitable for mucus penetration even in the absence of a desired mucolytic load. Therefore, ionic interaction promoting slow NAC release from SEDDS droplets was also required to achieve the optimal penetration of mucus, as mentioned earlier [14,15,16]. The early alterations in viscoelasticity upon NAC release from SEDDS, however, were connected to poor mucolytic activity in terms of improving overall diffusing particle mucopermeation, as seen below.

### 3.7. SEDDS Mucopermeation

**Figure 10:**
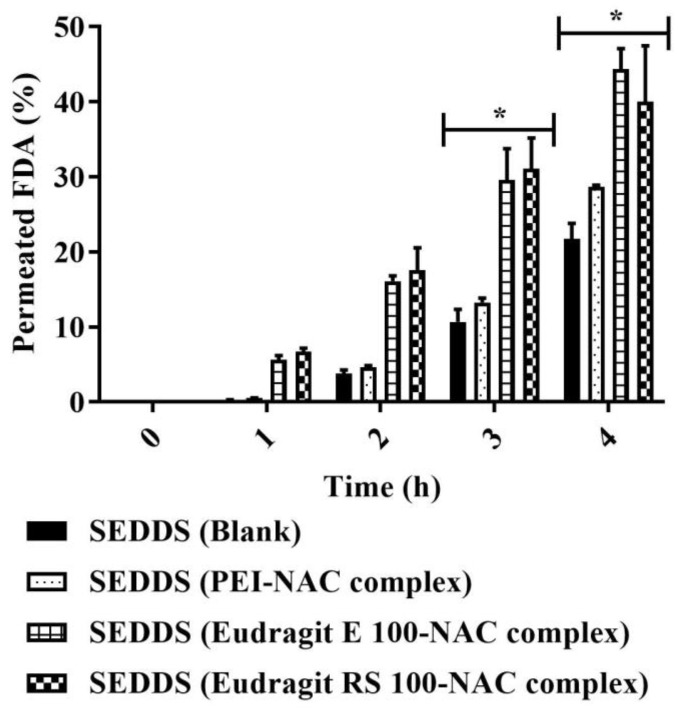
Transwell diffusion model assessing the amount of the diffused SEDDSs as a function of SEDDS FDA load that permeated the mucus gel layer over 4 h at 37 °C. By measuring FDA permeated percentage, the mucodiffusion comparison was drawn between the blank and mucoactive (loading different NAC polymer ionic complexes) SEDDSs. At 3 and 4 h, mucoactive SEDDSs had a significantly larger proportion of permeated FDA than blank SEDDSs (* *p* ≤ 0.05). Indicated values are the means ± standard deviations (*n* ≥ 3).

**Figure 11:**
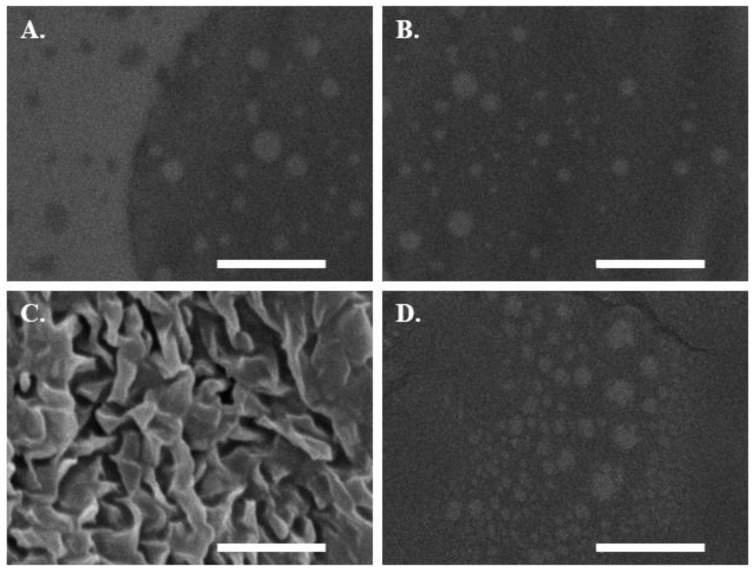
SEM images of the blank SEDDS (**A**) and mucoactive SEDDSs containing NAC complexes with PEI (**B**), Eudragit E 100 (**C**), and Eudragit RS 100 (**D**) permeated the mucus after 4 h incubation.

**Figure 12:**
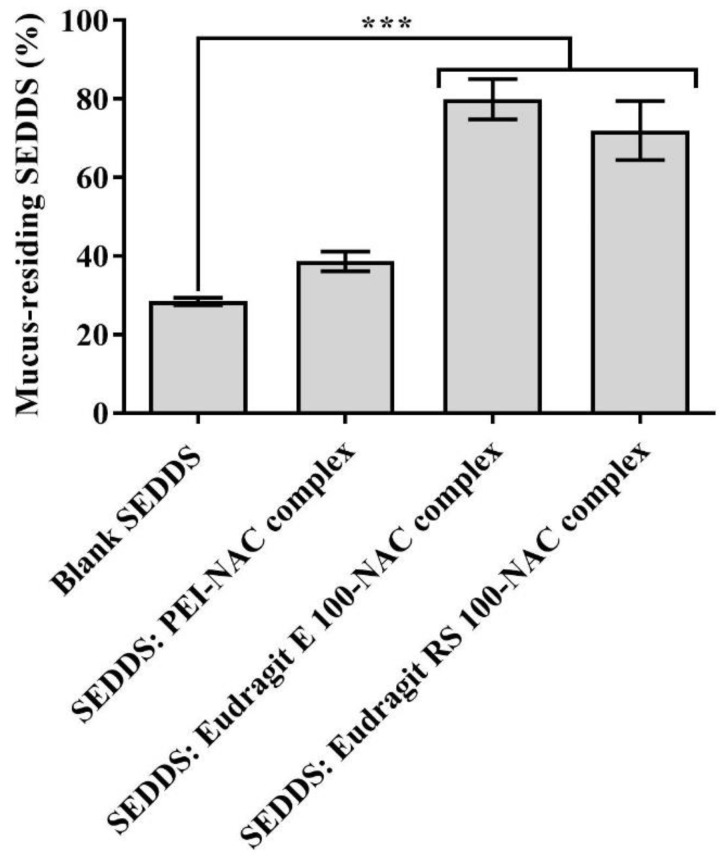
After 4 h of washing with 0.1 M phosphate buffer (pH 6.8) and incubation at 37 °C, the quantity of SEDDSs residing in porcine intestinal mucosal slices was measured using a 45° angle. *** *p* ≤ 0.001 versus a blank SEDDS. Indicated values are the means ± standard deviations (*n* ≥ 3).

SEDDS mucopermeation was addressed via the static in vitro transwell model by quantifying the amount of SEDDSs that diffused between two compartments, the donor and acceptor compartments separated by a mucus layer [9]. Figure 10 shows that mucoactive as compared with the blank SEDDS highlighted substantial increases in the amount of the loaded model drug FDA that permeated the mucus at all time points during an incubation period for 4 h. When Eudragit E 100– and RS 100–NAC complex-loaded SEDDSs were compared to the blank SEDDS, the percentage of diffused FDA from these two SEDDS complexes was up to 2-fold higher. The NAC content in the SEDDSs ranged from 0.5 to 1.5% (mass/vol.); nevertheless, there were no significant improvements in the amount of mucodiffused FDA in referral to PEI–NAC complex-loaded SEDDSs, the SEDDSs with the highest mucolytic load of NAC. In contrast, Eudragit E 100 and RS 100 complexes in SEDDSs, which had 1.5- and 3-fold lower NAC loadings, respectively, exhibited significantly greater mucodiffused FDA (*p* ≤ 0.05) than the control blank SEDDS. Therefore, mucoactive SEDDS reactivity to mucus to improve overall mucopermeation in this study correlated better with SEDDSs that slowly release their mucolytic load. In turn, this could explain why mucopermeation did not improve much for the PEI–NAC complex-loaded SEDDSs that exhibited immediate release of NAC. Due to the lack of sufficient lipophilic enhancement of free NAC in SEDDS droplets, a previously reported NAC load of 3% in SEDDSs showed minimal or no improvement in mucodiffusion. However, the same study reported equimolar novel thiol conjugates in SEDDSs namely, thiobutylamidine-dodecylamine (log P = 5.83) and thioglycolic acid-octylamine (log P = 3.14), with significantly improved mucodiffusion [16]. As a result, the mucoactive SEDDSs that steadily and gradually released their mucolytic load of lipophilic thiol conjugates had a greater impact on a well-established mucodiffusion as a result of a long-lasting mucolytic activity. Even though the NAC load varied among the integrated complexes within the SEDDSs, however, SEDDS diffusion via the mucus was no longer strongly prompted by the mucolytic NAC concentrations occurring in a narrow range in SEDDSs as compared with the effective mucolytic action of considerably higher NAC loads predicted to loosen overall mucus integrity. Overall, mucodiffusion was enhanced by the slower release rates of NAC properly lipidized as Eudragit E 100 and RS 100 complexes in SEDDSs. Slower-releasing SEDDS droplets resulted in more selective targeting of the surrounding mucus, resulting in physicochemical changes [15], and hence, increased the extent to which SEDDS droplets traversed the mucus. Clear evidence showing a greater abundance of Eudragit E 100-/RS 100-loaded as compared with PEI-loaded mucolytic SEDDS droplets that permeated mucus is provided in the SEM images shown in Figure 11A–D. Mucoactive SEDDSs with higher mucolytic performance were linked to significantly higher amounts of mucopermeating SEDDS droplets as compared with non-mucolytic SEDDSs. To further confirm the results of mucoactive SEDDS to which extent they permeated the mucus following the aforementioned transwell diffusion test, an ex vivo model assessing mucosal residence of the SEDDSs on dissected porcine intestinal segments was established. According to Figure 12, the amount of SEDDSs that remained resident on intestinal mucosa was linked in a direct relationship with their transportation capacity deeply through the multi-layered mucus. As compared with the blank SEDDS, the illustrated mucoresidence measurements indicated 2.8-, and 2.5-fold significantly (*p* ≤ 0.001) greater levels of mucoactive SEDDS residing in the intestinal mucosa after a 4 h incubation period, highlighting the mucolytic load of mucoactive SEDDSs containing Eudragit E 100–NAC, and Eudragit RS 100–NAC complexes, respectively. Unlike the lipophilic Eudragit E 100–NAC and RS 100–NAC complexes, the PEI–NAC complex’s SEDDSs as comparedwith the blank SEDDS exhibited a 1.3-fold higher amount of SEDDS droplets left on the intestinal mucosa that was non-significant (*p* > 0.05). This is because the immediately released NAC out of this SEDDS complex has non-significant mucolytic action and is washed away by the buffer medium’s continual flow. According to Leichner et al., using the mucolytic activity of lipidized papain enzyme ionically coupled with sodium deoxycholate and anchored in SEDDSs, the fraction of SEDDSs that remained resident on porcine intestinal mucosa was increased by twice as compared with unloaded SEDDSs. Using the transwell diffusion technique, they also demonstrated that increased mucosal residence of mucoactive SEDDSs was associated with a two-fold increase in SEDDS mucopermeation [48]. In this study, the enhanced intestinal mucosal residence corroborated the importance of lipophilic complexes in the investigation, as seen by mucoactive SEDDSs with improved mucolytic activity.

## 4. Conclusions

The size (<100 nm), PDI (<0.3), and zeta potential (average = −7.72 mV) stability among the produced mucoactive SEDDSs containing NAC polymer ionic complexes were all preserved in this study. The resazurin assay, which showed >85% cell viability, validated biocompatible and non-toxic SEDDS concentrations when applied on Caco-2 cells at 1% and 3% dilutions. SEDDSs demonstrated that NAC leaking out of droplets released at a significantly slower rate (*p* ≤ 0.001) from Eudragit RS 100 and Eudragit E 100 (log D > 5) as compared with PEI or free NAC (log D ≤ 0.5) that exhibited an immediate NAC release. Parallel to the NAC release behavior, intestinal mucus viscoelastic parameters influenced by the SEDDSs loading the NAC ionic complexes as compared with the blank SEDDS revealed considerably lower dynamic viscosity, elastic modulus, and viscous modulus. Finally, as compared with PEI–NAC complex-loaded SEDDS, mucoactive SEDDSs anchoring Eudragit E 100–NAC and RS 100–NAC complexes displayed significantly higher amounts of SEDDSs that permeated the mucus (*p* ≤ 0.05) and significantly improved SEDDS intestinal mucosal residence (*p* ≤ 0.001). Based on these findings, a significantly improved in vitro mucopermeation was achieved by mucoactive SEDDSs that showed adequate NAC sustained release. The resultant droplets of these SEDDSs were shown to produce better targeting mucolytic effect, meanwhile traversing mucus layers.

## Figures and Tables

**Table 1:** *N*-acetylcysteine target ionic complexes with selected cationic polymers.

*N*-Acetylcysteine	Polymer
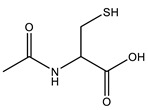 MW = 163.1 g/mol	**1. Non-quaternary amines:** 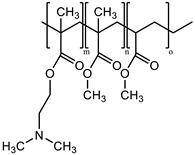 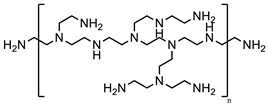 Eudragit E 100 (m:n:o = 1:2:1) Branched polyethyleneimine (25 kDa)
	**2. Quaternary amines:** 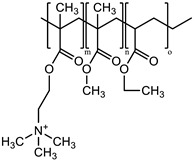 Eudragit RS 100 (m/n/o = 0.1:2:1)

**Table 2:** *N*-acetylcysteine (NAC) precipitation percentages found in ionic complexes with different selected polymers.

Polymers	MW (kDa)	Amino Substructures	Cationic Groups	(NAC/Polymer) Ratio	NAC (%) Precipitated in Complexes
Branched-PEI	25	Primary, secondary, tertiary	596	1: 1	74 ± 2.3
				1: 2	69.8 ± 2.4
Eudragit E 100	47	Tertiary	188	1: 1	55.9 ± 1.7
				1: 2	21.5 ± 0.7
Eudragit RS 100	32	Quaternary	10	4: 1	26.2 ± 0.7

## Data Availability

The data supporting the findings of the article are available within this article. All figures including the graphical abstract are original to this article and were neither imported nor reproduced from any other source.
